# Tensile and Viscoelastic Behavior in Nacre-Inspired Nanocomposites: A Coarse-Grained Molecular Dynamics Study

**DOI:** 10.3390/nano12193333

**Published:** 2022-09-24

**Authors:** Param Punj Singh, Raghavan Ranganathan

**Affiliations:** Discipline of Materials Engineering, Indian Institute of Technology Gandhinagar, Gandhinagar 382355, Gujarat, India

**Keywords:** nacre, biomimetics, nanocomposites, molecular dynamics simulations, mechanical behavior, viscoelasticity

## Abstract

Organisms hold an extraordinarily evolutionary advantage in forming complex, hierarchical structures across different length scales that exhibit superior mechanical properties. Mimicking these structures for synthesizing high-performance materials has long held a fascination and has seen rapid growth in the recent past thanks to high-resolution microscopy, design, synthesis, and testing methodologies. Among the class of natural materials, nacre, found in mollusk shells, exhibits remarkably high mechanical strength and toughness. The highly organized “brick and mortar” structure at different length scales is a basis for excellent mechanical properties and the capability to dissipate energy and propagation in nacre. Here, we employ large-scale atomistic coarse-grained molecular dynamics simulations to study the mechanical and viscoelastic behavior of nacre-like microstructures. Uniaxial tension and oscillatory shear simulations were performed to gain insight into the role of complex structure-property relationships. Specifically, the role played by the effect of microstructure (arrangement of the crystalline domain) and polymer-crystal interactions on the mechanical and viscoelastic behavior is elucidated. The tensile property of the nanocomposite was seen to be sensitive to the microstructure, with a staggered arrangement of the crystalline tablets giving rise to a 20–30% higher modulus and lower tensile strength compared to a columnar arrangement. Importantly, the staggered microstructure is shown to have a highly tunable mechanical behavior with respect to the polymer-crystal interactions. The underlying reasons for the mechanical behavior are explained by showing the effect of polymer chain mobility and orientation and the load-carrying capacity for the constituents. Viscoelastic responses in terms of the storage and loss moduli and loss tangent are studied over three decades in frequency and again highlight the differences brought about by the microstructure. We show that our coarse-grained models offer promising insights into the design of novel biomimetic structures for structural applications.

## 1. Introduction

**Figure 1:**
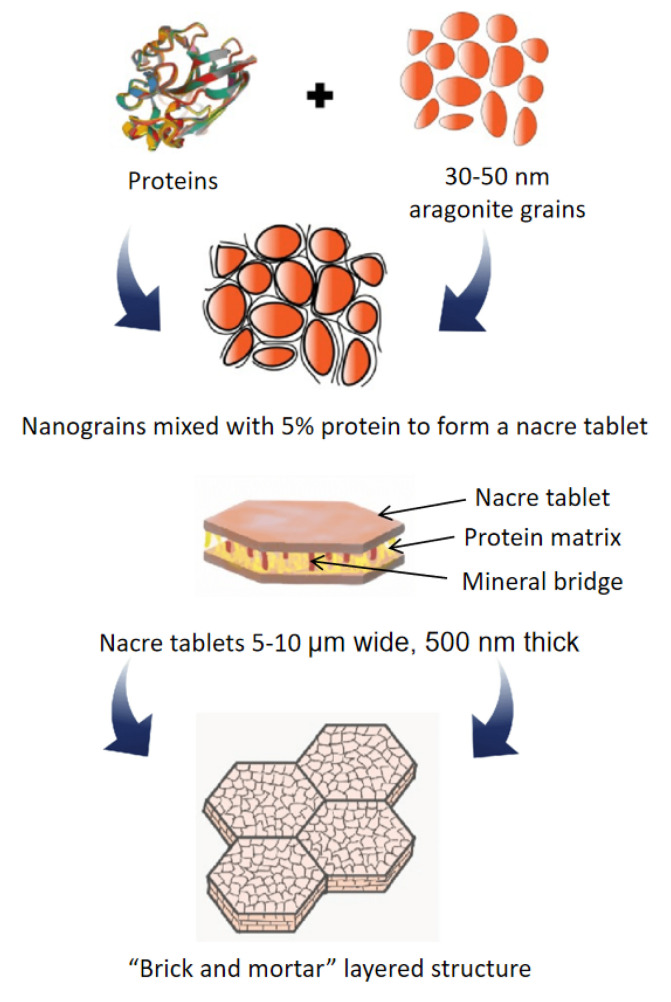
Schematic showing hierarchical brick and mortar layered structure of nacre at multiple length scales (Inspired from [7]). The Top left figure of a protein (chitin) has been adapted from the Protein Data Bank (PDB ID: 1ZTY) [8].

Our quest for a continual improvement in properties of high-performance materials has been kept alive thanks to novel structure-property relationships uncovered in natural materials. In recent years, the enhancement in the mechanical properties of materials has been bolstered by the structure and organization found in natural materials (nacre, bone, silk, etc.) holding excellent proficiency in creating complex configurations across different length scales. Nacre (known also as mother of pearl) is a classic example of hierarchical architecture that confers superior mechanical properties and has been the subject of biomimicry over the years. It forms the inner lining of mollusk shells and demonstrates a unique staggered composite architecture composed of about 95% aragonite, a crystallographic form of calcium carbonate, glued together with 5% of a biopolymer matrix [1]. Millions of aragonite nanocrystals of size 30–50 nm are mixed with proteins at nano-length scale to form aragonite tablets (an orthorhombic form of calcium carbonate) that are 10–20 μm wide and 0.5 μm thick. Interlocking aragonite tablets are staggered in successive laminae separated by a thin layer of protein resembling the hierarchical “brick and mortar” layered structure as shown in Figure 1. Furthermore, mineral protrusions known as mineral bridges (of the order of a few tens of nm) are distributed on the lamellar surface connecting adjacent tablets, forming a mechanical interlocking mechanism [2,3,4]. This architecture interacts synergistically in the nanometer and micrometer length scales; therefore, simultaneously enhancing stiffness, strength, and toughness, attracting a lot of attention in cutting-edge defense and aerospace applications [5,6].

The foundation of the macromechanical properties of nacre was established as early as in the 1970s through extensive tensile deformation studies [1,9]. More recently, several computational studies and models have been reported in the literature such as finite-element method simulations [10,11,12,13,14,15,16], micromechanical models [17], shear lag model [18,19] and tension-shear chain model [20]. These models have captured the respective role of hard mineral and their synergistic effect on the soft polymer to enhance mechanical stiffness, strength, and toughness of nacre’s layered structure very well. Most early studies reported elastic moduli are 70 GPa for dry nacre and 50 GPa for wet nacre [21], and tensile strength are 170 MPa (dry) and 130 MPa (wet) [9], respectively. The degree of hydration plays an important role in determining the stiffness and toughness; i.e., water acts as a plasticizer for organic molecules, resulting in higher failure strain and toughness. The fracture toughness of the nacre was measured to be 5–11 MPa/m${}^{2}$, which is approximately 10-fold higher than that of calcium carbonate [9]. Unlike dry nacre, which behaves similar to monolithic ceramic and undergoes brittle failure, the deformation behavior of hydrated nacre shows an initial elastic region till 70 MPa followed by interfacial yield through shear, generating local deformation. This phenomenon extends throughout the specimen and translates into a relatively larger strain at the macroscale. Once the entire shearing zone is exhausted, tablets start to be pulled out [22].

Over the past two decades, contrasting values of the tensile strength of the nacre have been reported, especially regarding loading along parallel and perpendicular directions with respect to the nacre tablets. The tensile strength parallels to tablet layers was estimated to be 150–200 MPa, whereas the strength along the perpendicular direction was reported to be 75–100 MPa, signifying the role played by the polymeric phase during deformation [23]. At multiple hierarchical length scales, synergistic effects such as interfacial strength and load transfer mechanisms between polymer and minerals have been recognized as important factors in determining the mechanical behavior of nacre. At the micro length scale, the enhancement in toughness is by virtue of inelastic viscoelastic and viscoplastic shear and crack deflection by the polymer matrix. Beyond elastic elongation, the soft protein matrix transfers the load to other crystalline platelets through the shear stretch, and rearrangement of chains leading to efficient energy dissipation [9,24,25].

The remarkable toughness of nacre is also attributed to the frictional resistance offered by nanoasperities [26,27] and theoretical strength of the mineral bridges [28] joining aragonite tablets present at a lower length scale. Atomic Force Microscopy (AFM), in conjunction with nanoindentation, has proven to be an effective tool for studying the underlying structural and mechanical characteristics of bio-nanocomposites at nano-length scale. Aragonite platelets at nano-length scales, which were long thought to be brittle, were revealed to be ductile through nanoindentation studies [29,30]. Various studies show that AFM images of plastic deformation at indented crack tip manifest the aragonite nanograins held with organic molecules as a building block of the nacre structure. In-situ Transmission Electron Microscopy (TEM) of nacre specimens under flexural load has shown the redistribution of stress facilitated by organic bridging between nanograins through rotation and sliding of grains in adding ductility to the system [31,32,33]. At the molecular level, the mechanical and viscoelastic behavior of polymer matrix has been investigated through AFM [34] and steered molecular dynamics simulations [35,36]. The force-displacement curve obtained from various experimental/computational methods of protein chains exhibits saw-tooth behavior due to bond-breaking and unfolding of proteins from the crystalline domain. Shorter protein molecules adhere to the mineral surface, increasing the strength locally. Once the ionic and covalent bonds are broken, the larger molecules demonstrate significant stretching. However, the protein molecules present as loops at the mineral interfaces enhance the area under the force-displacement curve. This is because more energy is needed to break the sacrificial bonds and unfold the looped chains. Hence, the type of polymer-mineral interaction or, in other words, the nature of bonding at the interface plays a crucial role in enhancing the mechanical properties of nacre-like composites [36,37].

Recent work on artificial ceramic/polymer nacre-like composites has shown remarkable enhancement in mechanical properties owing to the interlocked brick-mortar structure. Among these, graphene oxide/polymer [38] and Al${}_{2}$O${}_{3}$/polymer nacre-like composites [39,40] have exhibited up to a 4-fold increase in specific strength compared to nacre and other conventional composites of similar compositions, defying the rule of mixtures. Although most experimental studies were done on nacre and nacre-like materials are concentrated on quasi-static loading conditions; there is little understanding of nacre’s mechanical behavior under dynamic loading conditions. The studies are mostly concentrated on the nano-Dynamic Mechanical Analysis (DMA) test, with an initial focus on the microstructural aspect and the effect of loading frequency on nacre. Nano-DMA studies have shown that the loss moduli (*G*″) for nacre can be as high as 40 GPa at a frequency of 100 Hz and under a load of 1000 μN, proving the efficient damping nature of layered structures [41]. In another work, frequency-sweep performed through the nanoindentation experimental technique, reported significant energy-dissipation characteristics at 105 Hz. This led to the conclusion that the fracture toughness of the nacre increases with an increase in loading frequency [42].

With the advancement in computational resources, Molecular Dynamics (MD) simulations have become an indispensable and powerful tool to understand and predict the underlying mechanisms associated with a diverse class of artificial and natural materials. Owing to their tailorable properties, polymers and Polymer Nanocomposites (PNCs) have been comprehensively studied through MD simulations over the last decade [43,44,45,46,47,48,49]. It has been established that even a small addition of filler into the polymer matrix drastically alters the fundamental physics of polymers such as polymer chain relaxation and diffusion [46,47,48]. A significant change in strength, stiffness, and viscosity in PNCs has been observed owing to the synergistic effect between nanoparticles and polymer matrix. Recent work on multi-scale non-equilibrium tensile and oscillatory shear MD simulations has also provided deeper insight into the underlying deformation mechanisms due to the effect of the interfacial interaction strength, chemical grafting, coupling, cross-linking, filler volume fraction, and interfacial area, etc. [50,51,52,53,54]. Apart from conventional PNCs, there has been a significant rise in the study of bio-inspired polymer nanocomposites. Most notably, nacre-inspired layered structures have been in focus. Due to their hierarchical microstructure, chemical and physical interactions at the interface, and properties of the polymeric phase, which acts as a glue, significant enhancement in mechanical and viscoelastic properties of layered composites have been observed [55,56,57,58,59,60,61,62]. It is also noted that such layered polymer composites show the nanoconfinement effect [44,45,48,63] on polymers due to adjacent stiffer domains exhibiting a substantial change in viscoelastic properties of thin nanocomposite films [64,65].

Although the above studies on nacre-like composites and polymer nanocomposites have greatly enhanced our understanding of the mechanical and viscoelastic behavior of materials, the effect of microstructure and polymer-crystal interaction on the same for nacre-like composites remains unknown. Here, we try to provide new insights into the mechanical and viscoelastic properties of two coarse-grained models of nacre-like structures. We also compute the uniaxial tensile response, strain, and frequency-dependent dynamic moduli, elucidating the underlying deformation mechanisms. Finally, we show the effect of microstructure and polymer-crystal interaction that affects the polymer conformations and relaxations. Our coarse-grained models offer promising results pertaining to structure-property analysis. We speculate that our study may prove relevant in fine-tuning structure-property relationships and help design novel biomimetic architectures, eventually testing them under extreme conditions such as high-strain-rate impact and shock applications.

The rest of the paper is organized as follows. The simulation and post-processing methods for explaining the structure-property correlations are discussed in Section 2. Results and discussions on tensile and viscoelastic behavior are presented in Section 3, followed by conclusions of the study in Section 4.

## 2. Computational and Analysis Methods

All the simulations reported in this work were performed using the Large-scale Atomic/Molecular Massively Parallel Simulator (LAMMPS) package [66]. Visualization of configurations was performed using OVITO 3.5.4 [67]. Details of the coarse-grained model of polymer chains, crystalline domain, along with the simulation procedure are described in the following sections.

### 2.1. Model Systems

#### 2.1.1. Polymer Domain

Polymer chains were modeled using the Kremer-Grest bead-spring model [68]. The non-bonded beads interact with Lennard-Jones (LJ) potential, and the bonded interactions interact via the Finite Extensible Nonlinear Elastic (FENE) potential as shown in Equations (1) and (2), respectively.

(1) $$U_{L}{}_{J}=\{4\epsilon \left[{\left(\frac{\sigma }{r}\right)}^{12}-{\left(\frac{\sigma }{r}\right)}^{6}\right],r<2.5\sigma$$

$$\begin{array}{c} \;\{0\;,r>2.5\sigma \end{array}$$

where $U_{LJ}$ is the pair-wise LJ potential energy, *r* is the distance between two non-bonded beads, the LJ parameters ϵ is the depth of the energy well, and σ is the distance where the potential energy is equal to zero. The potential energy is truncated at 2.5σ.

(2) $$U_{FENE}=\{\frac{-KR_{0}^{2}}{2}\ln\left[1-{\left(\frac{r}{R_{0}}\right)}^{2}\right],r<R_{0}$$

$$\begin{array}{c} \;\{\infty \;,r>R_{0}, \end{array}$$

where *r* is the distance between bonded beads, $R_{0}=1.5\sigma$ is the maximum length of the bond at which potential diverges, and $K=30\epsilon /\sigma^{2}$ is the nonlinear spring coefficient.

#### 2.1.2. Crystalline Domain

**Figure 2:**
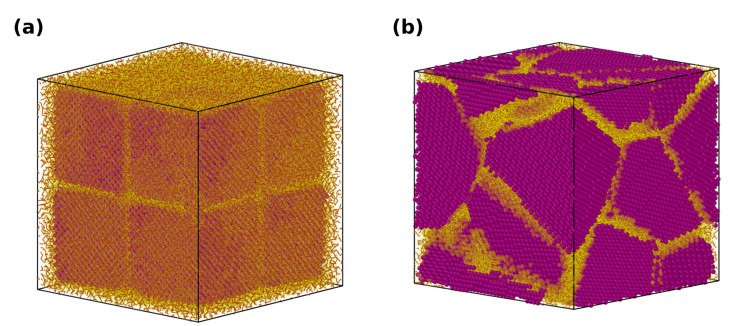
Equilibrated system snapshot of (**a**) columnar, (**b**) staggered nacre-like composites. Magenta and yellow represent the beads of crystalline domain and beads of polymer chains, respectively.

The crystalline domains in the nanocomposite structure were modeled as FCC crystallites described by the LJ potential. Two distinct morphologies were considered to mimic the *columnar* and *staggered* arrangement of crystallites in nacre (see Figure 2 for details). For the columnar morphology, eight cubic crystallites with length 27σ were stacked with a gap (about 6σ) uniformly around each crystallite along all three axes. These gaps were then filled with polymer chains prior to equilibration. For the staggered composite, a polycrystalline structure with an average grain size of 23σ was modeled using AtomsK [69]. Initially, only intragranular beads were linked through harmonic bonds. The simulation cell was allowed to expand to create gaps (of about 6σ) for the polymer domain, using a repulsive potential. Once the required gaps were created, the harmonic bonds were deleted, and polymer chains were inserted in the gaps.

#### 2.1.3. Creation of Composites

The two morphologies for nacre-like models, namely the columnar and the staggered structures, were constructed from the crystalline domains and polymer chains, modeled using the LJ and LJ + FENE potentials, respectively. A reduced unit system was used to represent all measurements. Each particle was represented as a bead of mass *m*. The $\epsilon_{p}$ for the polymer (soft domain) was set to unity, whereas $\epsilon_{c}$ for the crystalline domain (hard domain) was taken five times greater than that of the polymer to account for the contrast in the stiffness between the two domains. The interaction between polymeric and crystalline domains $\left(\epsilon_{cp}\right)$ was kept attractive and varied from $\epsilon_{cp}=1$ (weak interaction) to $\epsilon_{cp}=4$ (strong interaction) to model a range of interfacial strength between the two domains.

The total number of particles in the simulation cell was close to hundred thousand. Periodic boundary conditions (PBC) were applied along all the three axes. The columnar composite consists of monodispersed polymer chains, each with a chain length of 100 beads, inserted between the voids of crystalline domains. For the staggered structure; individual polymer beads were inserted in the gaps of the structure using the *“fix deposit”* command in LAMMPS, followed by an additional polymerization reaction by the creation of new bonds that link adjacent polymer beads present in the vicinity of one bond length (about 1.1σ) to form polydispersed chains. This was achieved using the *“fix bond/create”* command in LAMMPS, ensuring a mean chain length of about 100.9σ with a standard deviation of 34.17 beads, making the polymer chain statistics comparable with that of the columnar structure.

### 2.2. Equilibration Protocol

All simulations were performed with a time step of δt=0.001τ where τ is the dimensionless time unit, $\tau =\sigma \sqrt{\frac{m}{\epsilon }}$. To circumvent the initial unrealistic overlap of atomic positions due to the random placement of polymer chains, a soft repulsive potential was introduced to the polymer domain (while retaining the bonded terms), and the systems were run under the NVT ensemble for 100τ. Subsequently, the full LJ + FENE potential as described in Equations (1) and (2) was switched on with varying $\epsilon_{cp}$ for the composite systems. The structures were equilibrated in an NPT ensemble for 1000τ at the dimensionless constant pressure $P=0\epsilon /\sigma^{3}$ and temperature $T=0.2\epsilon /k_{B}$. The final structures were well below the polymer glass-transition temperature (see Section 3.1) with a reduced density of $\rho \approx 1.0m/\sigma^{3}$.

### 2.3. Non-Equilibrium Simulations

#### 2.3.1. Uniaxial Tension

To determine the tensile properties of the composites and to understand the role played by morphology and polymer-crystal interactions, uniaxial tensile deformation simulations were performed on equilibrated structures by deforming the simulation box along one axis while maintaining the pressure along the other two axes at $P=0\epsilon /\sigma^{3}$. The temperature was fixed at $T=0.2\epsilon /k_{B}$. Three separate samples were simulated for each $\epsilon_{cp}$ by deforming the box along the X, Y, and Z axes separately. The strain rate was fixed as $\dot{\epsilon }=0.001/\tau$. The Young’s modulus was computed from the linear region of the averaged stress-strain profile.

#### 2.3.2. Oscillatory Shear Simulation

The viscoelastic behavior of the composites was studied by applying an oscillatory shear deformation [48,49,63] and the resultant shear stress was analyzed. The equations of motion were integrated according to the SLLOD algorithm [70,71], which is equivalent to the Lees-Edwards “sliding brick” boundary conditions. The upper xy plane of the simulation cell was shifted parallel to the lower xy plane along the *x* axis so that each particle in the box has a “streaming velocity.” This velocity is subtracted from each particle’s velocity to yield a thermal velocity for computing the temperature and thermostat. During the oscillatory shear deformation, the shear strain imposed is governed by a sinusoidal function given by:

(3) $$\gamma_{xy}=\gamma_{0}sin\left(\omega t\right)$$

where $\gamma_{xy}$ is the oscillatory shear amplitude, and ω is the angular frequency. The virial shear stress [72] which is also a sinusoidal function, can be expressed as follows:

(4) $$\tau_{xy}=\tau_{0}sin\left(\omega t+\delta \right)$$

where $\tau_{0}$ is the shear stress amplitude and δ is the domain shift. The storage $\left(G^{\prime }\right)$, loss $\left(G^{\prime \prime }\right)$ moduli and the loss tangent (tanδ) are calculated as follows:

(5) $$\left(G^{\prime }\right)=\frac{\tau_{0}cos\left(\delta \right)}{\gamma_{0}}\;G^{\prime \prime }=\frac{\tau_{0}sin\left(\delta \right)}{\gamma_{0}}\;tan\delta =\frac{G^{\prime \prime }}{G^{\prime }}$$

The shear strain amplitude $\gamma_{0}$ was varied at a fixed frequency $f=0.01\tau^{-1}$ to study the viscoelasticity of the system as a function of shear strain. Non-Equilibrium Molecular Dynamics (NEMD) simulation for each system consists of at least 20 oscillatory shear cycles. The virial shear stress, $\tau_{xy}$, was recorded at an interval of every 10 time step. Strain amplitude in the linear viscoelastic regime was chosen for the study of frequency dependency of the dynamic moduli. NEMD simulations were carried out a angular frequency range of $0.0068\tau^{-1}$ to $15.7\tau^{-1}$, and the stress response was analyzed for at least 20 cycles.

### 2.4. Analysis

To gain insight into the dynamic behavior of glassy polymer chains in the affinity of the crystalline domain, we calculated the mean radius of gyration $R_{g}$, bond orientation parameter $<P_{2}>$, polymer-crystal coordination <Z>, Mean Square Displacement (MSD) and Rouse modes of relaxation.

#### 2.4.1. Radius of Gyration

It is essential to understand the molecular structural evolution of materials under tensile loading. During elongation, polymer molecules extend rapidly due to stretching. To analyze the deformation behavior of polymer chains, we studied the mean square radius of gyration $R_{g}$ as a function of strain. $R_{g}$ is calculated according to Equation (6), which represents the average size of a polymer chain [73].

(6) $$<{R_{g}}^{2}{>}^{1/2}=\frac{1}{N}<\sum_{n=1}^{N}{\left(r_{i}-r_{cm}\right)}^{2}{>}^{1/2}$$

Here, $r_{cm}$ is the position of the center-of-mass of the molecule and $r_{i}$ is the position of the i-th monomer for i=1,2,...,N. Here <> denotes the ensemble average of $R_{g}$ for all the chains present.

#### 2.4.2. Bond Orientation Parameter

Polymer chains are extended during tensile deformation and start orienting in the loading direction. The tensile stress arises from the loss of conformational entropy and increases in interaction enthalpy [74,75]. The bond orientation parameter $<P_{2}>$ contributes to the stress generated through conformational entropy. To measure this phenomenon, we computed the bond orientation parameter as a function of strain according to Equation (7) [76].

(7) $$<P_{2}>=\left(3<cos{}^{2}\theta >-1\right)/2$$

Here, θ is the angle between a given bond and the stretching direction (here, x-direction), and the notation <> represents the ensemble average of all the polymer chains in the system. For any system, $<P_{2}>$ may range from −0.5 to 1.0. A value of $<P_{2}>=-0.5$ indicates perfect perpendicular orientation to the loading direction. $<P_{2}>=0$ represents a random orientation, and a value 1.0 indicates perfect alignment parallel to the loading direction.

#### 2.4.3. Polymer-Crystal Interfacial Coordination Number

We studied the variation of the average coordination number <Z> of polymer-crystal interaction at the interface as a function of strain. <Z> is calculated as the area under the first prominent peak of the radial distribution function, g(r), to determine the average number of polymer beads adhered to the crystalline domain during the tensile deformation.

#### 2.4.4. Mean Square Displacement (MSD)

The mobility of polymer chains was investigated through computation of MSD of polymer domain according to Equation (8) [77].

(8) $$MSD=\langle \frac{1}{N}\sum_{i=0}^{N-1}{\left(r_{i}\left(t\right)-r_{i}\left(0\right)\right)}^{2}\rangle$$

where *N* is the number of beads, $r_{i}\left(t\right)$ and $r_{i}\left(0\right)$ is the position of bead at time *t* and time 0, respectively. MSD was calculated for varying $\epsilon_{cp}$ at the glassy state ($T=0.2\epsilon /k_{B}$).

#### 2.4.5. Polymer Chain Relaxation and Vibrational Density of States (VDOS)

The structural relaxations of polymer were investigated to consider the effect of polymer relaxation on the energy-dissipation characteristics of the nacre-like composites. The Rouse model describes the dynamics of polymer chains (of length *N*) at an intermediate length/time scale where the Rouse modes (p=0,1,2,...,N−1) corresponds to distinct internal relaxations. For our study, the Rouse modes were computed as per Equation (9) [63,78].

(9) $$\vec{X_{p}}={\left(\frac{2}{N}\right)}^{\frac{1}{2}}\sum_{i=1}^{N}\vec{r_{i}}cos\left[\left(\frac{p\pi }{N}\right)\left(i-\frac{1}{2}\right)\right]$$

where $r_{i}$ denotes the normal coordinates of the particle. The autocorrelation function of Rouse modes (calculated according to Equation (10)) is expected to decay exponentially, are generally independent of each other, and provide an estimate of the likely relaxation times for the polymer chains.

(10) $$\langle \vec{X_{p}}\left(t\right).\vec{X_{p}}\left(0\right)\rangle =\langle {\vec{X_{p}}}^{2}\rangle e^{-\frac{t}{\tau_{p}}}$$

To compute the Rouse modes, a homopolymer system of N=100 chain length was simulated under NVT conditions at a melt temperature $\left(T=1.0\epsilon /k_{B}\right)$ as well as in the glassy $\left(T=0.2\epsilon /k_{B}\right)$ state. The particles’ coordinates were recorded at an interval of 10 time steps during a long production run of $10^{7}\tau$. The autocorrelation functions of Rouse modes were calculated using the Fast Fourier Transformation (FFT) algorithm [79].

It is well known that in the harmonic approximation, the power spectrum of the velocity correlation is the vibrational density of states as per Equation (11) [49,80].

(11) $$f\left(\omega \right)=F\left(\frac{<\sum {\vec{v}}_{i}\left(0\right){\vec{v}}_{i}\left(t\right)>}{<\sum {\vec{v}}_{i}\left(0\right){\vec{v}}_{i}\left(0\right)>}\right)$$

where the sum is taken for the number of atoms taken for the analysis. The power spectrum generally yields peaks corresponding to the fundamental frequencies and gives the VDOS of the materials.

## 3. Results and Discussion

This section is divided into three subsections. The first presents results of characterization of the glass transition and polymer chain statistics of the composites. This is followed by two subsections on (a) tensile and (b) viscoelastic properties of the composites.

### 3.1. Glass-Transition Temperature and Polymer Chain Statistics

**Figure 3:**
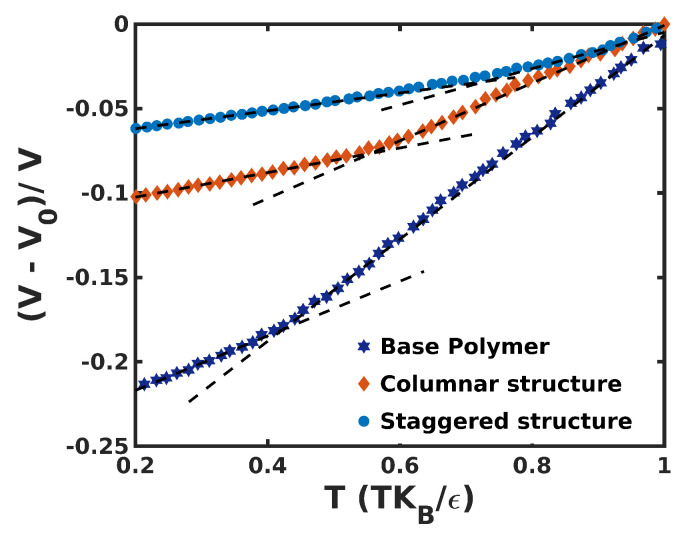
Plot of the system volume as a function of temperature during cooling for the homopolymer and composite systems.

To investigate the effect of the microstructure on the glass-transition temperature, $T_{g}$ of the composites, we simulated the quenching of the composites from $T=1.0\epsilon /k_{B}$ to $0.2\epsilon /k_{B}$ under the NPT ensemble at a cooling rate of $8*10^{-6}\tau^{-1}$[81]. The volume of the systems was monitored continuously as a function of temperature, where *V* and $V_{0}$ represents the volume of the system at the final and initial time step, respectively. Notably, two different slopes were observed, and the temperature at which the transition between two slopes occurred was considered to be the glass-transition temperature $\left(T_{g}\right)$. Figure 3 compares the simulation results of variation in volume as a function of temperature for the composite systems.

To validate our results, $T_{g}$ of the base polymer consisting of 300 chains with 100 beads each was calculated and found to be equal to 0.42$\epsilon /k_{B}$, consistent with the literature [81]. Furthermore, the composites showed a significant enhancement in $T_{g}$, with the columnar structure and the staggered structure showing the $T_{g}=0.55\epsilon /k_{B}$ and $T_{g}=0.72\epsilon /k_{B}$, respectively. The increase in $T_{g}$ of the composites was likely due to the high loading of crystallites that hindered the motion of polymer chains in the confined spaces. However, the staggered structure introduced a larger barrier to the polymer mobility, therefore imparting the segmental relaxation of polymer chains over a considerable range of temperatures. On the other hand, the smooth surfaces of the crystallites present in the columnar structure permit easier chain sliding and comparatively faster relaxation, resulting in a reduced $T_{g}$.

The conformation of polymer chains, defined by their equilibrium end-to-end distance $R_{ee}$ and the radius of gyration $R_{g}$, are usually associated with the relaxation and stiffness of chains. Specifically, large polymer conformations extend the relaxation time and thus affect the $T_{g}$ and viscoelastic properties. To check this effect, we computed the average end-to-end distance $<R_{ee}>$ of polymer chains to observe the effect of polymer conformation in determining $T_{g}$ of the system, and the results are enumerated in Table 1 We infer that the composites have a two-fold higher mean $R_{ee}$ compared to the base polymer, whereas the staggered composite shows a significant difference (about 14%) in mean $R_{ee}$ in comparison to the columnar structure.

### 3.2. Tensile Properties of Composites

**Figure 4:**
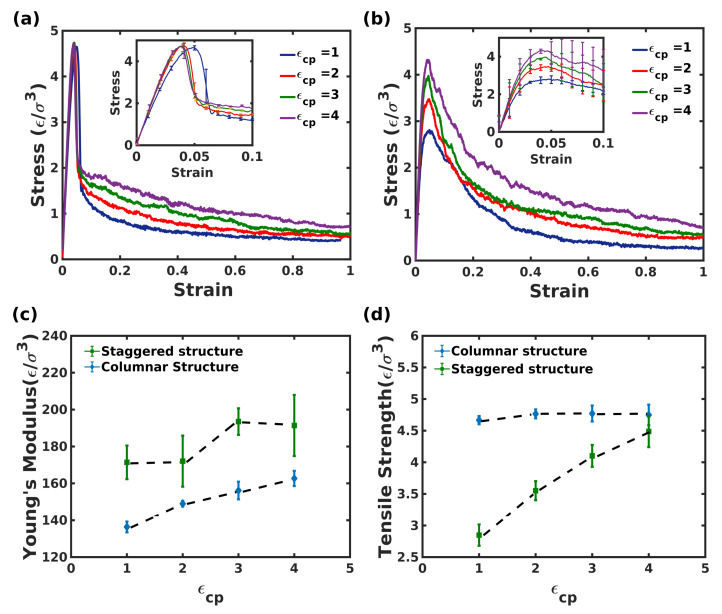
Stress-strain response (**a**) columnar structure for different polymer-crystal interactions $\left(\epsilon_{cp}\right)$, (**b**) staggered composite for different $\left(\epsilon_{cp}\right)$, (**c**) Young’s modulus for columnar and staggered structure and (**d**) Tensile strength for columnar and staggered composite for different $\left(\epsilon_{cp}\right)$. The plot contains the standard deviation calculated during tensile deformation along the X, Y, and Z axes. Dotted lines are guidelines for visualization.

Figure 4 compares the stress-strain behavior and mechanical properties of the columnar and the staggered composites under uniaxial extension, for various values of the polymer-crystal interaction strengths, $\epsilon_{cp}$.

It is apparent that the composites exhibit an initial elastic regime followed by the yield point at strain ≈4 to 5%. The simulation results are comparable to the results obtained from the tensile extension of nacre [33]. It is important to highlight that the initial high stiffness is due to the load carried predominantly by the stiffer crystalline domains with insignificant contributions from the polymer domains. Moreover, the effect of microstructure on the tensile behavior is quite different for the two composites. Although insignificant changes to Young’s modulus and tensile strength were observed for the columnar structure, the staggered composite featured a monotonic rise in Young’s modulus and a nominal increase in the elastic limit with $\epsilon_{cp}$. An $\epsilon_{cp}=4$ shows a 12% increase in the modulus compared to $\epsilon_{cp}=1$.

**Figure 5:**
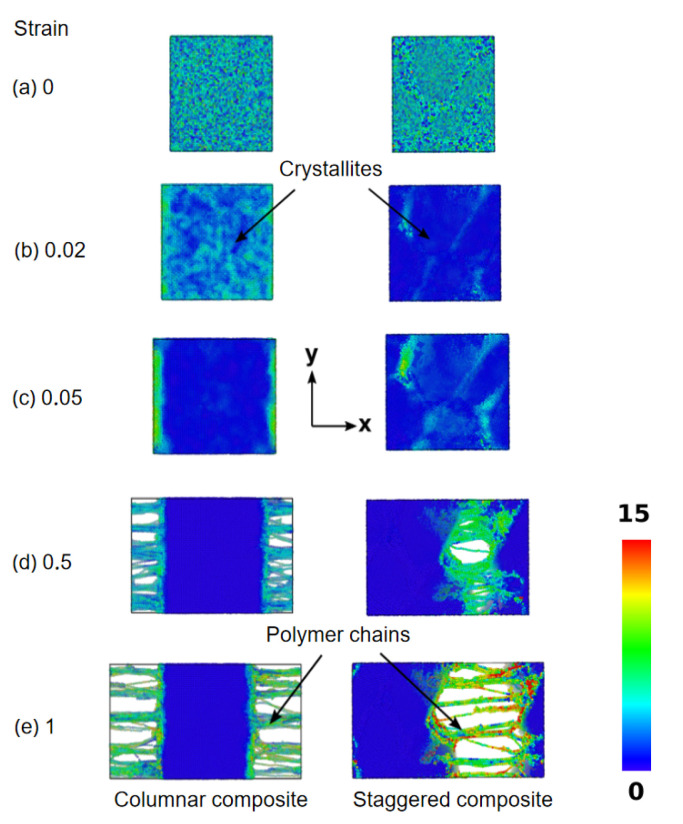
Snapshots of tensile deformation of nacre-like composites for increasing strain. Color coding represents the shear strain in the system at particular elongation.

Interestingly, the negligible effect of $\epsilon_{cp}$ on the tensile properties of the columnar structure was confirmed by the deformation trajectory of the composite systems as shown in Figure 5. It was observed that after the initial elastic expansion, the chains present on the crystallite surface sustained the load predominantly, as opposed to the highly adsorbed chains present in the mid-sections joining the crystallites of the columnar composite. The polymer chains present on the surface were easily pulled irrespective of the polymer-crystal interaction, owing to the periodic arrangement of the crystallites as shown in panels (d) and (e). Post-yield point, the stress decreased abruptly in the case of columnar structure and gradually in the case of the staggered structure, depending on the load carried through molecular mechanisms such as polymer chains slippage, molecular rearrangements, and delamination of polymer chains from the crystallites. The strain hardening effect, which is typically observed in nacre, was absent for both structures, as the only mode of resistance against extension is the polymer-crystal interaction strength, $\epsilon_{cp}$. Thus, rapid slippage and delamination of polymer chains account for the decrement in the stress response for the composites. A much higher toughness (25.25% higher for $\epsilon_{cp}=4$) for the staggered structure is due to a greater ability of the polycrystalline-like crystallites to resist the slippage and delamination of polymer chains even up to 20% strain. It is thus clear that polymer-crystal interaction and the microstructure play an important role in determining the mechanical behavior of nacre-like structures and, by extension, for various structural motifs found in biomaterials.

#### Mechanisms Responsible for Tensile Behavior

**Figure 6:**
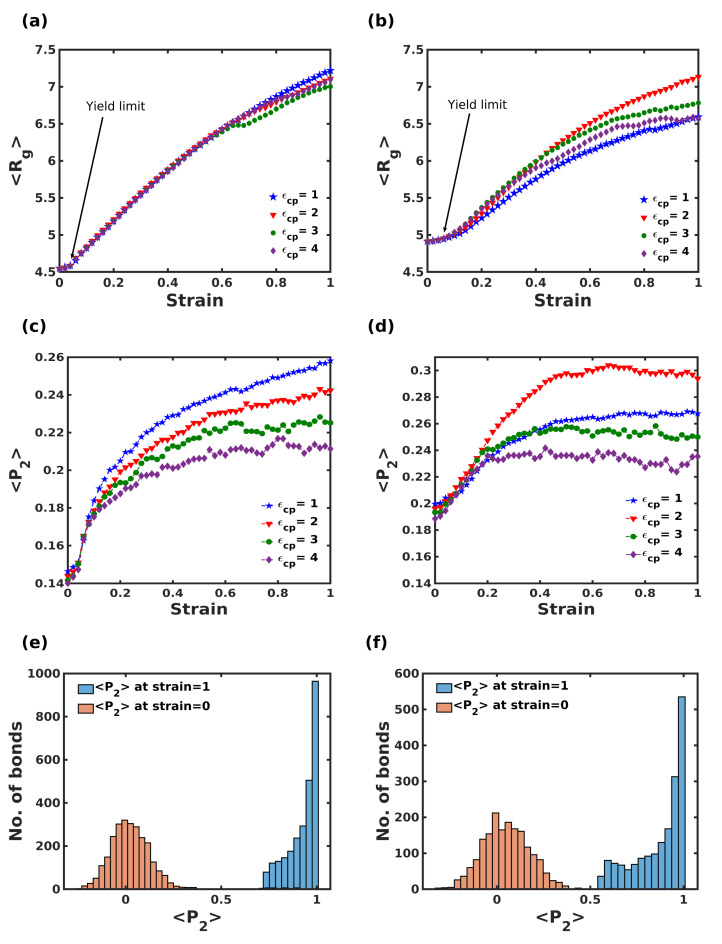
Mean radii of gyration $<R_{g}>$ and bond orientation parameter $<P_{2}>$ plot of (**a**,**c**) columnar, (**b**,**d**) staggered composite for different polymer-crystal interactions as a function of strain. (**e**,**f**) show the histograms of bond orientation parameter of polymer chains (20% of chains that underwent large inelastic extension) at strain = 0 (orange) and 1 (blue).

When subjected to extension, polymer chains exhibit morphological changes in response to the load. The conformational changes to polymer chains during extension are dependent on the conformational entropy to a large extent. To quantify this effect, the average radius of gyration $<R_{g}>$ and bond order parameter $<P_{2}>$ were calculated as a function of tensile strain as shown in Figure 6.

A monotonic increment in $<R_{g}>$ and $<P_{2}>$ were exhibited for the polymer chains beyond the elastic regime. As mentioned earlier, in response to the increasing strain, the polymer chains present on the surface normal to the loading direction contributed to significant morphological changes and are mostly independent of $\epsilon_{cp}$ for the columnar composite. On the contrary, the variation in $R_{g}$ is remarkably influenced by the polymer-crystal interaction strength for the staggered structure, where higher $R_{g}$ corresponds to weaker $\epsilon_{cp}$. From panels (c) and (d), it is noteworthy to see the small quantitative change in $<P_{2}>$ that directly corresponds to the minor variation in the conformational entropy of the polymer chains in the glassy state during extension. As the overall population of polymer chains is trapped in the cage-like interface, the significant changes to the conformational entropy were believed to be due to the fraction of polymer chains undergoing major inelastic extension. Through the tensile deformation plot shown in Figure 4, it was clearly seen that a fraction (≈20%) of polymer chains underwent a large inelastic extension and was completely reoriented in the loading direction during expansion. To illustrate this fact, we calculated the $<P_{2}>$ parameter for the fraction of the chains involved in the extension at strain ϵ=0 and ϵ=1. Panels (e) and (f) show the histogram of $<P_{2}>$ parameter for the fractional chains undergoing huge inelastic deformation. The results proved that these chains were randomly oriented $<P_{2}>=0$ at zero strain (as shown in orange bars), and these chains eventually reoriented along the loading direction $<P_{2}>=1$ for the complete extension.

**Figure 7:**
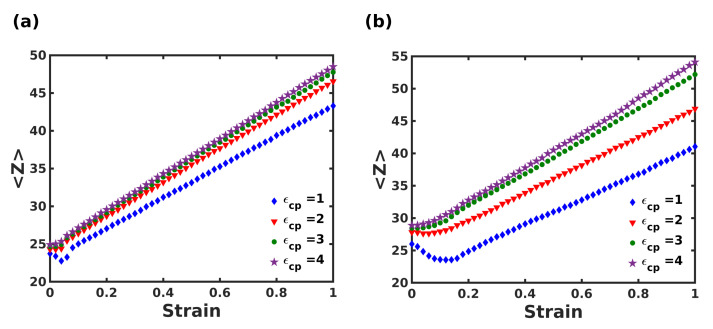
Plot of polymer-crystal coordination number at the interface as the function of strain for (**a**) columnar, (**b**) staggered composite for varying polymer-crystal interactions.

Additionally, the polymer-crystal coordination number <Z> at the interface was computed as a function of strain during extension and is shown in Figure 7. Both composite systems showed a linear increase in <Z> with strain. A small decrease in <Z> was observed for low interaction strengths, up to strain corresponding to the elastic regime. This was due to the initial separation of polymer beads from the crystallites. On the other hand, strongly adsorbed polymer chains ($\epsilon_{cp}$ = 3 and 4) showed a monotonic increase in <Z>. It is noteworthy that the columnar composite exhibited insignificant variation in <Z> for varying $\epsilon_{cp}$, whereas the staggered composite exhibited highly tunable mechanical behavior with respect to the interfacial interaction strength.

### 3.3. Viscoelastic Behavior

Under non-equilibrium conditions, the viscoelastic behavior depends on various deformation conditions, most importantly the strain amplitude and the frequency of deformation, which are explored in the following sections. The nature of interactions between the crystalline and polymeric domains also naturally impact viscoelasticity and are studied in the following sections.

#### 3.3.1. Effect of Strain Amplitude

**Figure 8:**
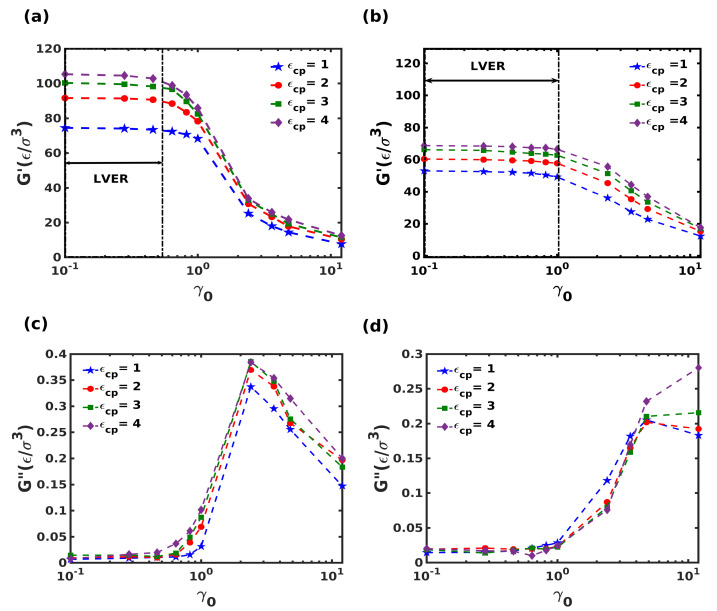
(**a**,**b**) Storage modulus (*G*${}^{\prime }$) and (**c**,**d**) loss modulus (*G*″) for different polymer-crystal interactions, as a function of shear amplitude for the columnar (panels (**a**,**c**)) and the staggered (panels (**b**,**d**)) composite systems.

Oscillatory shear simulations were performed by varying the shear strain amplitude from $0.1\gamma_{0}$ to $10\gamma_{0}$ at a constant frequency of $0.01\tau^{-1}$ and the variation of the storage modulus (*G*${}^{\prime }$), and loss modulus (*G*″) are shown in Figure 8.

Beyond the Linear Viscoelastic Region (LVER), the polymer dynamics change from glassy to rubbery state due to the segmental relaxation of the composites, manifested by the transition in *G*${}^{\prime }$ and *G*″ as a function of shear strain amplitude. A closer inspection of the transition regime indicated the delayed relaxation of polymer chains in the staggered structure over a wide range of shear strain amplitude (≈150% higher strain amplitude). The sharper decline of *G*${}^{\prime }$ of the columnar composite is consistent with the lower $T_{g}$ of the columnar structure. Additionally, storage and loss moduli are sensitive to the polymer-crystal interaction strength $\epsilon_{cp}$. As shown in Figure 8, higher $\epsilon_{cp}$ leads to higher storage and loss moduli for both composites. This is not surprising since a larger interaction strength facilitates better stress transfer between the crystalline and polymeric domains, leading to larger overall stiffness. We observe contrasting behaviors in loss modulus for the two composites at strain amplitudes of $1.0\gamma_{0}$, with the columnar structure showing a monotonous decrease in *G*″ with strain, whereas the staggered structure shows a further increase in *G*″ for larger $\epsilon_{cp}$, especially after strain amplitude of $4.0\gamma_{0}$, showing the tunable nature of damping in the staggered composite, akin to the tensile behavior.

#### 3.3.2. Effect of Deformation Frequency

**Figure 9:**
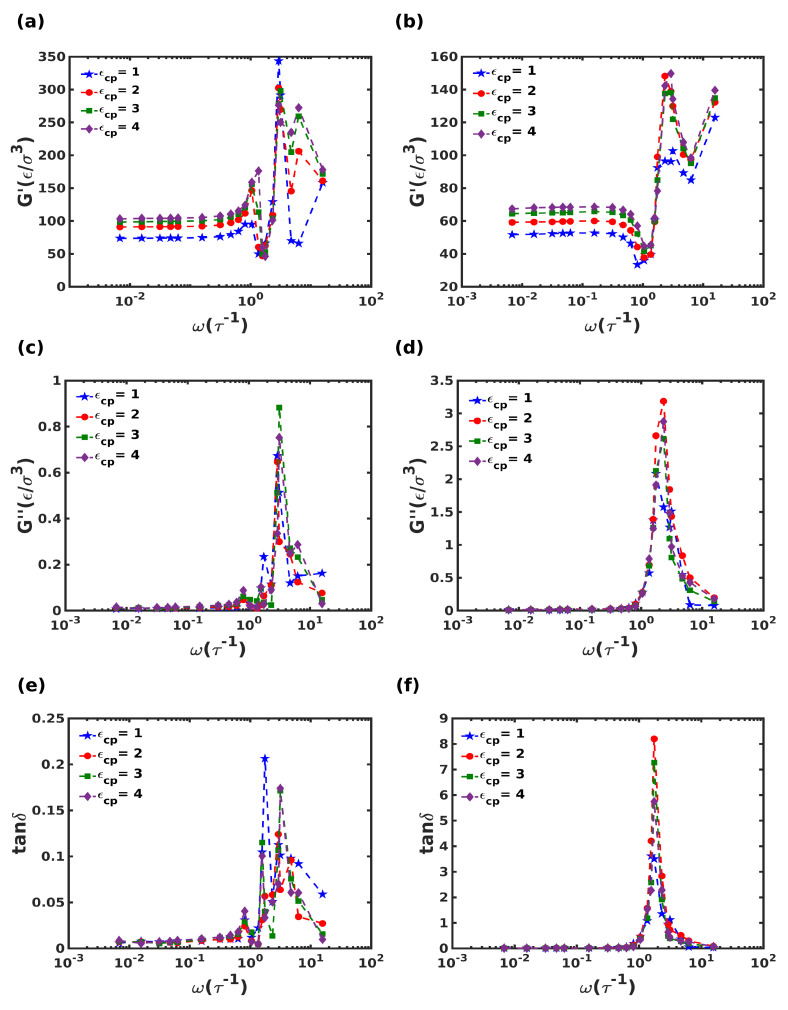
(**a**,**b**) Storage modulus (*G*${}^{\prime }$), (**c**,**d**) loss modulus (*G*″), and (**e**,**f**) tanδ for different polymer-crystal interactions, as a function of shear frequency for the columnar (panels (**a**,**c**,**e**)) and the staggered (panels (**b**,**d**,**f**)) composite systems.

The dependence of dynamic moduli on the oscillatory deformation frequency was studied by varying the frequency, ω from $0.0068\tau^{-1}$ to $15.7\tau^{-1}$ to give a wide frequency spectrum spanning more than three decades, commonly referred to as the *frequency-sweep* test. The shear amplitude was fixed at $\gamma_{0}=0.5\%$, within the LVER. The storage and loss moduli and the loss tangent, (tanδ) are plotted for both composites in Figure 9.

First, at low frequencies, $0.0068\tau^{-1}<\omega <1\tau^{-1}$ the systems have sufficient time to respond to the oscillatory deformation. Correspondingly, for both systems, the *G*″ is negligible in comparison to *G*${}^{\prime }$ in the frequency range spanning up to the two decades of frequency. Under this frequency range, the *G*${}^{\prime }$ values for the columnar and the staggered structure were found to be in the range of $70-100\epsilon /\sigma^{3}$ and $50-70\epsilon /\sigma^{3}$, respectively. It has also been observed that the composites exhibited a 6−8 fold rise in *G*${}^{\prime }$ compared to the pristine polymer $\left(G^{\prime }=7\epsilon /\sigma^{3}\right)$. Here, an increase in the interaction strength between the crystalline and polymeric domains led to a monotonic increase in *G*${}^{\prime }$.

At intermediate frequencies, namely between $1\tau^{-1}<f<10\tau^{-1}$, characteristic peak(s) appear in the *G*${}^{\prime }$ and *G*″ plots as seen in panels (a–d). The staggered composite showed 3 times higher G" value than the columnar composite exhibiting tunable viscoelastic properties with respect to the microstructure. Interestingly, the characteristic peak(s) in *G*${}^{\prime }$ and *G*″ for the systems clearly propound the synergistic action among the crystallites and the polymer. The combined effect of crystallite vibration and stiffness enhancement in the polymer at high frequency led to an enhancement in *G*${}^{\prime }$.

The variation of tanδ with frequency is shown in panels (e–f), demonstrating a 40-fold increase in the loss tangent for the staggered structure compared to the columnar composite. In comparison, the peak tanδ for the homogeneous polymer phase was found to be 6.7 (not shown here). For the staggered composite, an increase in $\epsilon_{cp}$ exhibited a reduction in the loss tangent (or alternatively, an enhancement in the damping characteristics). Multiple peaks were observed for the columnar composite at frequencies in the range of ω=1.75 to $3.14\tau^{-1}$. In contrast, the staggered composite exhibited a single loss peak at $\omega =2.32\tau^{-1}$. The stark differences in the damping behaviors for the two structures arise from morphological differences in the two structures, especially regarding the orientation of the polymer chains; in the columnar structure, all polymer chains occupy voids that are along the three principal axes, whereas the staggered composite contains chains oriented along random voids throughout the system.

Finally, a significant rise in the storage modulus was observed at much higher frequencies in the range $10\tau^{-1}$ to $11\tau^{-1}$. This enhanced stiffness of the composites with an increasing frequency usually indicates the frozen oscillatory motion of the particles at small time scales.

#### 3.3.3. Domain-Dependent Stress Response

**Figure 10:**
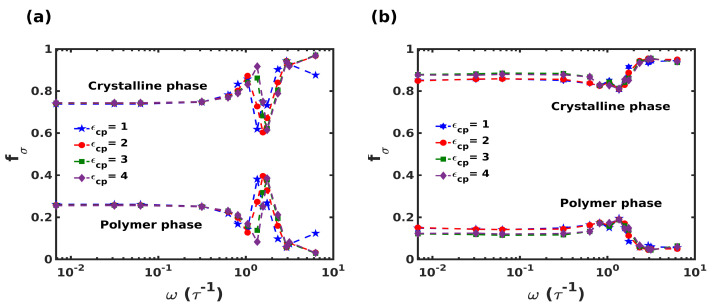
Stress fraction $\left(f_{\sigma }\right)$ of crystalline and polymer domain for (**a**) columnar composite and (**b**) staggered composite varying polymer-crystal interactions, respectively.

To characterize the stress-strain response during the oscillatory deformation further, the distribution of shear stress in each domain (i.e., crystalline and polymeric domains) is plotted as a function of frequency in Figure 10. The stresses in the domains are represented as the fraction of total stress, $\left(f_{\sigma }\right)$, sustained by the domain. The stress values for individual components were computed by considering the peak stress generated at a particular frequency. The striking aspect is the significant fraction of stress borne by the polymeric domain. For frequencies in the range $0.0068\tau^{-1}<\omega <1\tau^{-1}$, the polymeric domain carried around 20% of the overall stress. The most interesting part is the dramatic shift in the stress-carrying contribution at the frequency corresponding to the peak observed in loss moduli. At this frequency, the polymeric domain supported 40% of the stress in the columnar composite, whereas only a small increment was observed in the case of the staggered composite. Another significant point observed was the effect of polymer-crystal interaction $\epsilon_{cp}$ on the stress response. It was seen that in the columnar composite, for smaller $\epsilon_{cp}$, the stress response peak shifted towards lower frequencies, which is in accordance with the multiple relaxation peaks observed during frequency-sweep simulations reported in Section 3.3.2. In contrast, a negligible effect of $\epsilon_{cp}$ was observed for the staggered structure.

#### 3.3.4. Mechanisms Responsible for Viscoelasticity

(a) Polymer chain dynamics

**Figure 11:**
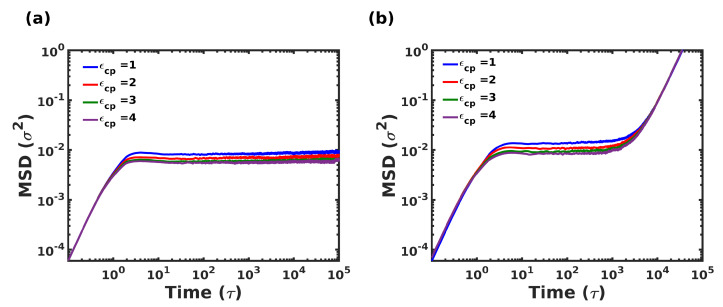
Mean Square Displacement (MSD) as the function of time at equilibrium at $T=0.2\epsilon /k_{B}$ for (**a**) columnar, (**b**) staggered composite systems.

One of the dominant dissipative mechanisms in polymer nanocomposites is the frictional forces that arise from the relative sliding of polymer chains and the sliding between the polymer and filler. Although traditional nanocomposites contain filler fraction in the range 10–40%[82,83], our biomimetic composites are completely opposite in design, with the polymeric phase accounting for just 20% by volume. Thus, it is important to characterize the dynamics of polymer chains present in confinement, and this was characterized by calculating the MSD of the polymer chains in equilibrium. Figure 11 shows the variation of MSD in both composites with respect to time during a long NVT simulation, for various $\epsilon_{cp}$.

As expected, the presence of crystallites dramatically slowed down the overall dynamics of the polymer chains. Typical solid-like behavior was observed for the polymeric domain at the temperature $T=0.2\epsilon /k_{B}$. Under confinement, all the polymer chains were strongly adsorbed to the crystallites, with negligible contribution due to $\epsilon_{cp}$. A slight effect was observed at intermediate times, where lower interaction strength exhibits higher mobility as expected. In contrast to the columnar composite, the staggered structure exhibits slightly greater displacements, albeit only within 1σ, which too is at times greater than $10^{4}\tau$.

(b) Rouse mode analysis

**Figure 12:**
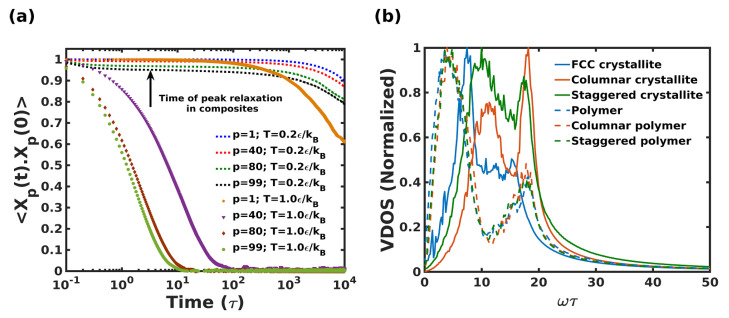
(**a**) Rouse mode numbers denoted (p=1,40,80 & 99) for homopolymer system of chain length N=100 at melt $\left(T=1.0\epsilon /k_{B}\right)$ and glassy state $\left(T=0.2\epsilon /k_{B}\right)$. The autocorrelation function is normalized to scale the characteristic time scale, (**b**) VDOS of the composite systems added to partial VDOS of components arising from the crystalline and polymeric domains.

The lack of polymer mobility was also corroborated by the computation of the Rouse relaxation times and its possible role in energy dissipation. It was thought that the segmental dynamics of polymers might be affected due to high crystallite loading, suggesting a possible correlation between the frequency of deformation (or its inverse, the time period) at maximum energy dissipation (which corresponds to the peak observed in the loss moduli) and the relaxation times of Rouse modes. Therefore, to verify the hypothesis, Figure 12a compares four characteristic Rouse modes of the homogeneous polymer system at two temperatures corresponding to the melt (T = 1$\epsilon /k_{B}$) and glassy states (T = 0.2$\epsilon /k_{B}$). Specifically, the four modes, *p* = 1, 40, 80 and 99, were compared.

The Rouse mode analysis was quite revealing in several ways. First, polymers at the melt condition showed a well-defined exponential decay characterizing the Rouse time for the individual modes. In contrast, at the glassy state, polymer dynamics were found to be substantially slower, with negligible segmental relaxation. This is a direct consequence of the highly confined polymeric domain and rules out the direct correlation of polymer relaxation to that of the peak dissipation time period, which is in the order of 1τ–10τ of the frequency-sweep simulation (indicated by a black arrow in Figure 12a).

(c) Vibrational Density of States

The observation of peak damping in the high-frequency regime (as seen in Figure 9) strongly hints at the role played by the Vibrational Density of States (VDOS) of the composites. To check for this effect, Figure 12b shows the VDOS calculated for various systems, namely homogeneous FCC crystallite, and homogeneous polymer, followed by the partial VDOS arising from the crystalline and polymeric domains in both the composites. For the composite systems, $\epsilon_{cp}$ was fixed at 2.3, the geometric mean of $\epsilon_{c}$ and $\epsilon_{p}$. A clear correlation of the peak loss frequency with the polymer VDOS frequency between ω= 0–$10\tau^{-1}$ in the composites was observed. The prominent peak in the polymer VDOS corresponds to the inter and intra non-bonded LJ terms, and the weaker peak at higher frequencies corresponds to bonded FENE interactions [84,85]. It was also interesting to note the shift in the VDOS peaks of the crystallites toward higher frequencies in the composites in comparison to the VDOS of the homogeneous FCC crystallite. This shift is attributed to the combined effect of crystallite stiffness and enhanced stiffness of the confined polymer-crystal interface at higher frequencies. This shows that the mechanism of high-strain rate viscous dissipation in nacre-like composites, especially below $T_{g}$, is likely due to the *anharmonic coupling of vibrational modes* as described in prior literature [46], rather than any relaxation-related or dynamical aspects of the polymeric domain.

## 4. Conclusions

This study set out to investigate the tensile and viscoelastic properties of nacre-like composites for two different microstructures, namely columnar and staggered. The role played by several design factors and deformation conditions such as the microstructure, the polymer-crystal interaction, deformation frequency, and amplitude, etc., were elucidated through non-equilibrium coarse-grained molecular dynamics simulations. Some of the significant findings are:

1. The mechanical properties of biomimetic, nacre-like composites can be highly tunable for certain morphologies such as the staggered composite, especially as a function of the polymer-crystal interaction strength $\epsilon_{cp}$. The Young’s modulus, and the tensile strength increased with higher $\epsilon_{cp}$.
2. Conformational analysis of polymer chains during inelastic deformation exhibited the significant role played by $\epsilon_{cp}$ on the deformation behavior of composites. The weakly adsorbed polymers on the crystallites exhibited notable deformation as verified by $<R_{g}>$ and $<P_{2}>$ parameters during tensile deformation.
3. The rough crystallite surface in the staggered composite dramatically arrests and delays the dynamics of polymer chains in the vicinity of the crystallites, exhibiting a notable increment in the glass-transition temperature. This, in turn, affects mechanical behavior.
4. A detailed study of viscoelastic properties of the composites indicated a 150% increment in the LVER for the staggered composite in comparison to the columnar structure. Additionally, it was observed that $\epsilon_{cp}$ plays a significant role in the stiffness and dissipative characteristics of the staggered composite.
5. The dissipative behavior of the nacre-like composites is very sensitive to the deformation frequency and can be tuned by tuning the microstructure. The maximum loss tangent for the staggered composite was found to be 8, which was 40 times higher than that for the columnar structure, which in turn is 34 times lower with respect to the homogeneous polymer.
6. At frequencies corresponding to maximum damping, about 15–30% of the overall stress was supported by the polymer domain.
7. The dynamics of polymer chains were substantially restricted in the cage-like confined regions. The mean square displacement and Rouse mode analysis of polymeric chains essentially showed a solid-like behavior, corroborating its highly confined nature. Finally, the large damping effects, especially at high deformation frequencies in nacre-like composites, are a direct consequence of the vibrational properties of the constituent atoms.

We show ultimately that there are some molecular-level phenomena such as the vibrational density of states and polymer-crystal orientation effects that affect the viscoelastic and tensile properties, respectively, while other phenomena such as polymer diffusivity and relaxation do not play a role in the deformation characteristics, given that the polymeric domain is in a glassy state. Thus, macroscopic properties have direct molecular underpinnings. These results add to the rapidly expanding field of structure-property correlation of high-performance biomimetics. It is envisaged that these simulation techniques can be easily extended to model various biomimetic microstructures found in nature for optimization of mechanical properties such as strength, toughness, damping, and even high-strain rate deformations such as impact [86,87] and shock loading [88,89], therefore guiding the experimental design of such materials. Moreover, qualitative structure-property relationships elucidated in this work can be extended to other hierarchical structures found in nature, such as bones, silk, wood, etc. [90,91,92,93,94,95], providing an important tool for designing future high-performance materials.

## Figures and Tables

**Table 1:** Mean end-to-end distance $R_{ee}$ with standard deviation for the equilibrated base polymer and composite systems.

System	$T_{g}$	Mean $R_{\mathrm{ee}}$ (σ)	Std. ($R_{\mathrm{ee}}$)
Base polymer	0.42	25.26	8.94
Columnar composite	0.55	48.01	15.37
Staggered composite	0.72	54.62	20.14

## Data Availability

The data presented in the study are available on request from the corresponding author.
